# MyPEEPS Mobile App for HIV Prevention Among Transmasculine Youth: Adaptation Through Community-Based Feedback and Usability Evaluation

**DOI:** 10.2196/56561

**Published:** 2024-05-30

**Authors:** Dorcas Adedoja, Lisa M Kuhns, Asa Radix, Robert Garofalo, Maeve Brin, Rebecca Schnall

**Affiliations:** 1 Columbia University School of Nursing New York City, NY United States; 2 Ann & Robert H. Lurie Children's Hospital of Chicago Chicago, IL United States; 3 Department of Pediatrics Feinberg School of Medicine Northwestern University Chicago, IL United States; 4 Callen-Lorde Community Health Center New York, NY United States

**Keywords:** HIV, mobile app, transgender men, transmasculine

## Abstract

**Background:**

Transgender men and transmasculine youth are at high risk for acquiring HIV. Growing research on transgender men demonstrates increased HIV risk and burden compared with the general US population. Despite biomedical advancements in HIV prevention, there remains a dearth of evidence-based, sexual health HIV prevention interventions for young transgender men. MyPEEPS (Male Youth Pursuing Empowerment, Education, and Prevention around Sexuality) Mobile is a web-based app that builds on extensive formative community–informed work to develop an evidence-based HIV prevention intervention. Our study team developed and tested the MyPEEPS Mobile intervention for 13- to 18-year-old cisgender young men in a national randomized controlled trial, which demonstrated efficacy to reduce sexual risk in the short term—at 3-month follow-up. Trans men and transmasculine youth resonated with basic HIV educational information and sexual scenarios of the original MyPEEPS app for cisgender men, but recognized the app's lack of transmasculine specificity.

**Objective:**

The purpose of this study is to detail the user-centered design methods to adapt, improve the user interface, and enhance the usability of the MyPEEPS Mobile app for young transgender men and transmasculine youth.

**Methods:**

The MyPEEPS Mobile app for young transgender men was adapted through a user-centered design approach, which included an iterative review of the adapted prototype by expert advisors and a youth advisory board. The app was then evaluated through a rigorous usability evaluation.

**Results:**

MyPEEPS Mobile is among the first mobile health interventions developed to meet the specific needs of young transgender men and transmasculine youth to reduce HIV risk behaviors. While many of the activities in the original MyPEEPS Mobile were rigorously developed and tested, there was a need to adapt our intervention to meet the specific needs and risk factors among young transgender men and transmasculine youth. The findings from this study describe the adaptation of these activities through feedback from a youth advisory board and expert advisors. Following adaptation of the content, the app underwent a rigorous usability assessment through an evaluation with experts in human-computer interaction (n=5) and targeted end users (n=20).

**Conclusions:**

Usability and adaptation findings demonstrate that the MyPEEPS Mobile app is highly usable and perceived as potentially useful for targeting HIV risk behaviors in young transgender men and transmasculine youth.

## Introduction

Transgender men and transmasculine youth are at high risk for acquiring HIV. Growing research on transgender men demonstrates increased HIV risk and burden compared with the general US population. A recent review was among the first to systematically estimate HIV risk and burden in transgender men in the United States. In this review, there was a high prevalence of HIV (3.2%; laboratory confirmed) and high rates of unprotected sexual intercourse (24.5%), which has been strongly associated with HIV acquisition [1]. High rates of sex work (13.1%) were also reported in the review, and research suggests that the marginalization of sex work increases the likelihood of seroconversion [2]. Previously undescribed HIV risk behaviors and contextual factors point to the immediate need for the development and testing of HIV prevention interventions among transgender men, particularly younger transgender men and transmasculine youth who may be at an increased risk due to vulnerability associated with developmental age and increasing sexual activity.

Despite biomedical advancements in HIV prevention, there remains a dearth of evidence-based, sexual health HIV prevention interventions for young transgender men. The current Centers for Disease Control and Prevention compendium of evidence-based interventions (EBIs) for HIV prevention has no EBIs for young transgender men [3]. In response to this need, our study team sought to adapt MyPEEPS (Male Youth Pursuing Empowerment, Education, and Prevention around Sexuality), a theoretically driven intervention for young transgender men. The MyPEEPS intervention was originally developed for cisgender young men who have sex with men and targets social-cognitive and cognitive behavioral factors based on best practices for behavior change, emotion regulation, and HIV intervention (eg, knowledge, self-efficacy, and behavioral skills) within young men who have sex with men–specific social contexts [4]. MyPEEPS scenarios include emotionally activating and cognitively complex situations involving partner-specific factors (eg, older partners), sexualized contexts (eg, web-based sexual partner interaction), and experiences of social stigmatization (eg, by race or sexual orientation). The scenarios also specifically address emotional regulation and minority stress, which are salient issues facing adolescents at risk for acquiring HIV due to structural inequality. Our study team developed and tested the MyPEEPS Mobile intervention for those aged 13-18 years in a national randomized controlled trial, which demonstrated efficacy to reduce sexual risk in the short term—at 3-month follow-up [5]. MyPEEPS Mobile is a web-based app that builds on extensive formative community–informed work to develop an evidence-based HIV prevention intervention [4,6-8].

However, the MyPEEPS Mobile interventions were not developed specifically for young transgender men. We explored adaptation to the specific needs of young transgender men inclusive of their unique underlying mechanisms of sexual risk and HIV acquisition. We conducted focus groups with 49 young transgender men at the 4 MyPEEPS Mobile sites to achieve this goal, which generally supported the usefulness of the content but highlighted important limitations requiring adaptation. Limitations related to specific health concerns (eg, pregnancy prevention and disclosure), body parts, body types, and sexual partner dynamics were raised [9]. The purpose of the study described herein was to build on our prior success with the MyPEEPS Mobile intervention and fill a gap in HIV prevention interventions by adapting MyPEEPS Mobile to young transgender men and transmasculine youth aged 15-25 years, which is an age group that captures a period of increasing risk of HIV infection [10]. We also aimed to assess the usability of the app among the target population. This paper outlines key user-centered design methods to adapt and improve the interface and usability of the MyPEEPS Mobile app for young transgender men and transmasculine youth.

## Methods

### Study Design

**Figure 1:**
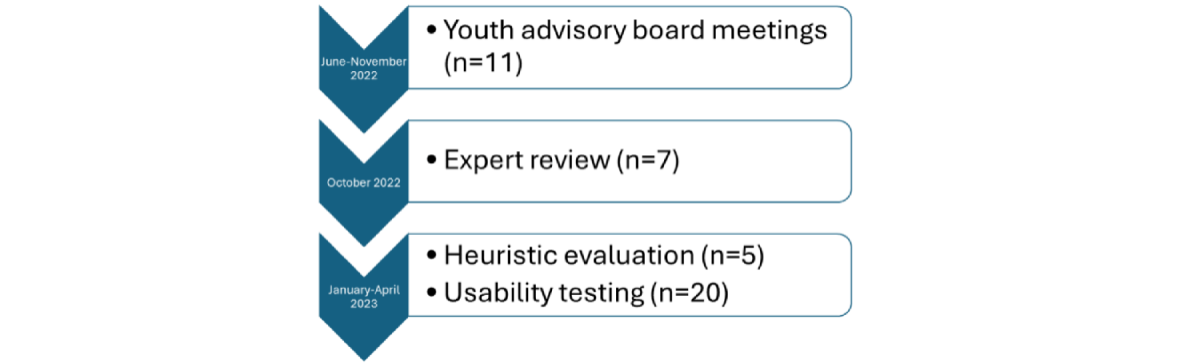
Timeline of data collection.

The MyPEEPS Mobile app for young transgender men and transmasculine youth was adapted through a user-centered design approach for mobile health (mHealth) apps [7,11,12], which included an iterative review of the adapted prototype by expert advisors and a youth advisory board (YAB) from June to November 2022. The app was then evaluated through a rigorous usability evaluation from January to April 2023. The details of the timeline for each phase of the study are illustrated in Figure 1.

### Ethical Considerations

All study activities were reviewed and received approval by the Columbia University institutional review board (IRB-AAAT8624). Study participants provided written informed consent. All electronic data were stored in a certified environment. All study data were stored on secure HIPAA (Health Insurance Portability and Accountability Act)–compliant servers at the Columbia University Medical Center campus. All study data were password protected and were maintained in a completely secure and HIPAA-compliant environment. YAB members received US $75 for each session in which they participated. Experts received US $250 for reviewing the curriculum. Usability testing participants received US $40 Amazon gift codes for their time, and experts who participated in the heuristic evaluation received US $150 Amazon gift codes.

### Youth and Expert Advisory Board Review

#### Sample

To adapt the MyPEEPS Mobile app, we recruited expert advisors and end users. Expert advisors included those with expertise in mHealth app development, adolescent health, sexual health, and gender minority youth health who were identified by the investigative team. The YAB included transgender men and transmasculine youth between the ages of 18 and 24 years, who reported condomless sex with partners assigned male at birth. The YAB members were considered consultants, and the research team had the final say in all adaptations. While the research team made the final determination about the app content, YAB members who reviewed the finalized curriculum expressed unanimous approval. The recruitment letter was circulated to community-based organizations, and a recruitment flyer was shared via social media platforms to recruit end users, using a convenience sampling approach.

#### Procedures

Both groups reviewed a pencil-and-paper prototype of the intervention content for the salience of the narrative content, flow of modules, and features and characteristics of the app activities and characters. Expert advisors provided feedback by email in 2 iterative rounds. The YAB reviewed the app content, images, and activity structure of the original MyPEEPS Mobile app, as well as initial adaptations and new activities proposed by the expert advisory group to optimize salience to users. The YAB met 10 times to review the paper-and-pencil prototype for the app over a period of 5 months (June-November). They reviewed all activities, covering 3-5 activities in each of the initial 5 sessions. Extensive notes were recorded in each meeting, and the prototype was then revised and reviewed in weekly study team meetings. In the final 5 meetings, YAB members reviewed the revised prototype and responded specifically to outstanding questions. In between meetings 9 and 10, the expert advisory group provided input on the prototype and it was revised accordingly. In the final meeting of the YAB, these additional revisions were reviewed to produce a beta version of the app for usability testing.

### Usability Testing

#### Sample

**Figure 2:**
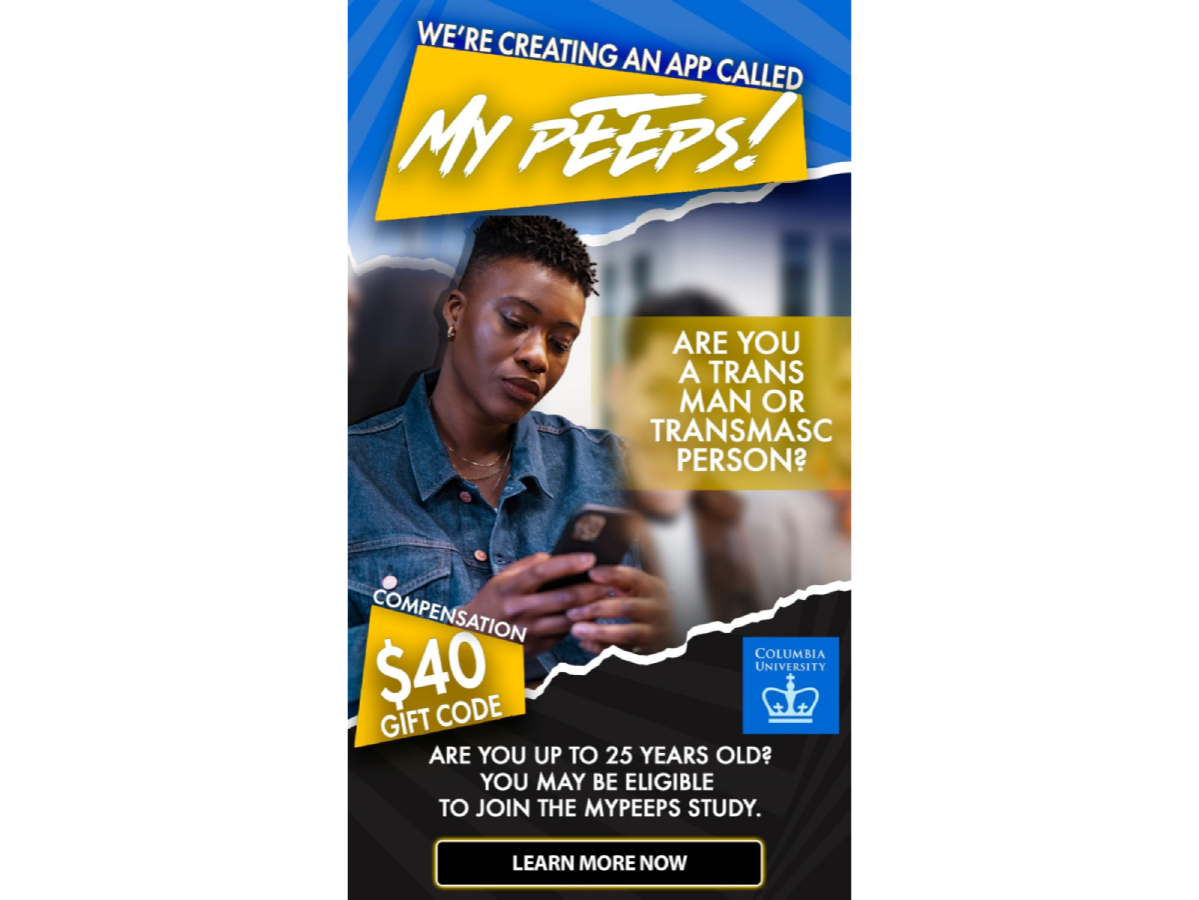
Sample study recruitment material. MyPEEPS: Male Youth Pursuing Empowerment, Education, and Prevention around Sexuality.

End users were eligible if they (1) were aged 15-25 years; (2) assigned female sex at birth; (3) self-identified as a man, transgender man, or transmasculine; (4) understood and read English; (5) lived in the United States; (6) owned a smartphone; (7) self-reported condomless receptive anal or vaginal penile sex with a partner assigned male at birth in the past year; and (8) self-reported HIV negative or unknown status. To recruit participants for the usability testing, we paid to poststudy advertisements on social media platforms including Instagram and Facebook. The lead research coordinator also shared information about the study on Discord and Lex, which are networks their transmasculine identity allowed them to access with community trust. Promotional materials were also shared with community-based organizations. A sample study advertisement that was approved by our institutional review board and posted on social media sites is illustrated in Figure 2.

#### Procedures

Interested participants completed a screener through REDCap (Research Electronic Data Capture; Vanderbilt University) to determine whether they were eligible for the study based on the inclusion criteria detailed above. Once determined to be eligible for usability testing, end users completed usability testing procedures over the Zoom conferencing platform (Zoom Technologies Inc) with the research coordinator (DA). Following the completion of written informed consent, participants reviewed the adapted MyPEEPS Mobile app content, images, and activity structure to identify usability issues and improve the overall content and functionality of the MyPEEPS Mobile app for young transgender men. Participants were asked to open the MyPEEPS Mobile app and complete 13 use cases (Textbox 1) while using the app. Participants were instructed to “think aloud” while navigating through the app and completing tasks. Notes were taken by the study coordinator to record feedback and assess the ease or difficulty of completing each task.

At the end of the visit, participants were asked to complete a survey through Qualtrics XM. Participants self-reported demographic information and completed the Health Information Technology Usability Evaluation Scale (Health-ITUES) and the Post-Study System Usability Questionnaire (PSSUQ) to assess the usability of the MyPEEPS app. The Health-ITUES is a 20-item scale that consists of 4 subscales: impact (items 1-3), perceived usefulness (items 4-12), perceived ease of use (items 13-17), and user control (items 18-20). Each item is rated between 1 (strongly disagree) and 5 (strongly agree), with a higher score indicating a more usable tool. The PSSUQ is a 16-item scale divided into 3 subscales: system usefulness (items 1-6), information quality (items 7-12), and interface quality (items 13-16). Each item is rated between 1 (strongly agree) and 7 (strongly disagree), with a lower score indicating a more usable tool. Participants were compensated US $40 in the form of an Amazon gift code for completing usability testing.

**Use cases for testing MyPEEPS (Male Youth Pursuing Empowerment, Education, and Prevention around Sexuality) young transgender men app to assess usability.**

1. Open the MyPEEPS app
2. Log in to the MyPEEPS app
3. MyPEEPS profile setup
4. Complete the Bulls-I activity
5. Complete the BottomLine activity
6. Complete P’s On-Again BottomLine activity
7. Complete the Move Up, Move Back activity
8. Complete the Jeopar-T activity
9. Complete the Tackling Testing activity
10. Complete the Safer Injection activity
11. Complete the Disclosure and Safety activity
12. Complete the Red Flag, Green Flag activity
13. Log out of the account

### Heuristic Evaluations

#### Sample

Our sample comprised experts trained in human-computer interaction and had published in the field of informatics. Participants were identified by the principal investigator (RS) and were invited to participate via email invitation.

#### Procedures

Heuristic evaluations took place over Zoom and consisted of the completion of 13 use cases (Textbox 1), a survey, and 3 open-ended questions. Open-ended questions were (1) Thinking back to the MyPEEPS Mobile app, how do you think transmasculine youth who want to prevent HIV exposure would apply the information, lessons, or activities in their daily lives? (2) How do you perceive this app would be of relevance to transmasculine youth who want to prevent HIV exposure? and (3) How easy or intuitive is it to navigate through the app to perform a particular task? Zoom visits were audio recorded as experts remotely tested the app interface and provided feedback. Participants were compensated US $150 in the form of an Amazon gift code.

In the survey, usability experts rated the app according to Nielsen’s 10 heuristics: (1) visibility of system status; (2) match between system and real world; (3) user control and freedom; (4) consistency and standards; (5) help users recognize, diagnose, and recover from errors; (6) error prevention; (7) recognition rather than recall; (8) flexibility and ease of use; (9) aesthetic and minimalist designs; and (10) help and documentation. Each heuristic was rated between 0 (not a usability problem) and 4 (usability catastrophe) [13].

## Results

### YAB and Expert Advisors

A total of 11 YAB members provided feedback on curriculum adaptations, with an average of 4 participants attending each meeting. Most of the YAB members (n=7, 64%) were recruited via social media. The YAB was diverse in terms of race and ethnicity. YAB participants identified as Black (n=4, 36%), White (n=5, 45%), and biracial (n=2, 18%). A small minority of YAB members also identified as Hispanic or Latino or Latinx (n=2, 18%). The mean age of the YAB members was 23 (SD 3; range 21-24) years and were largely from the Northeast (n=7, 64%), with the balance of the group from the South (n=4, 36%) and the West Coast (n=1, 9%).

The original MyPEEPS Mobile app included 21 mobile app activities [6]. The revised app was adapted for young transgender men and includes 25 total activities. Most of the activities and the original focus of the app on social-cognitive and cognitive behavioral factors were maintained in the adapted version. The primary adaptations for the underlying mechanisms of risk unique to young transgender men are listed in Table 1. These can be summarized as changes to MyPEEPS characters (look and feel); minor wording changes within the activities for specificity to young transgender men; and major changes, including removing, expanding, or adding activities.

More background information for MyPEEPS characters was added to the Underwear Personality Quiz, a “warm-up” activity to familiarize app users with the characters (activity 4). The characters were also given ages, racial backgrounds, and hobbies to increase user identification with the characters. Updates were made to their pronouns, gender, romantic identity, and sexuality descriptions as well. One character was made physically larger to reflect variation in physical body sizes, and skin blemishes were added to characters so they appeared more like adolescents.

Minor changes included updates to common phrases and lingo, adding more current emojis, and including information about gender, gender expression, anatomy, and sexual behaviors relevant to young transgender men and transmasculine youth throughout the app. A resource page with a list of trans-affirming social and health organizations was developed, including resources specifically for transgender men and transmasculine youth. Hyperlinks to these resources were included throughout the app as well.

Major changes to activities included removing 1 activity called “Rubber Mishap” that was viewed by the YAB as more appropriate for a younger age group (ie, too “silly” for this age group). Five activities originated from another app designed by members of the team for young transgender women (LifeSkills Mobile [14]), including “Jeopar-T” (activity 10), “Safer Injection” (activity 16), “Disclosure and Safety” (activity 18), “Red Flag, Green Flag” (activity 19), and “Healthy Relationships” (activity 21). These activities were adapted for young transgender men and added to the MyPEEPS app. For example, Jeopar-T, which is a game to identify fact from fiction as it relates to health topics, was adapted to discuss health topics relevant to young transgender men including potential pregnancy, birth control options, and gender-affirming hormone therapy. The Safer Injection activity was adapted to focus on correct injection steps for injectable testosterone. Disclosure and Safety focuses on safer disclosure of transgender status to sexual and romantic partners. Red Flag, Green Flag is an activity focused on critical appraisal of potential web-based sexual and romantic partners, and Healthy Relationships is an activity to describe and reinforce characteristics of healthy sexual and romantic relationships.

In addition, several activities were revised or expanded for young transgender men, including the following activities: “Bulls-I” (activity 2), “Sexy Settings” (activity 6), “HIV True/False” (activity 9), and “Tackling Testing” (activity 13). The original purpose of the Bulls-I activity was to create awareness of the different social roles held by everyone as a precursor to goal setting; however, in this adaptation, we repurposed the graphic and concept to prompt users to identify their own body parts by name. This allowed for a more in-depth and affirming discussion of sexual behaviors. Sexy Settings was adapted for an older age group by including information about statutory consent and public sex laws as well as more scenarios involving sex work. The HIV True/False activity was updated with more detailed information on pre-exposure prophylaxis (PrEP) and a set of prompts to motivate PrEP adherence (for those individuals taking PrEP). Tackling Testing (originally called, Testing with Tommy) was adapted to maintain the original purpose (demystify HIV testing) and also to address other health concerns among young transgender men and transmsasculine youth, including pregnancy potential and PrEP access.

**Table 1:** Primary adaptations of the MyPEEPS^a^ Mobile app to address the underlying mechanisms of risk unique to young transgender men .

Activity	Status	Purpose	Adaptation
1. MyPEEPS Profile Set Up	Original	Profile setup	Added option for users to type in or select their pronouns Added a feature allowing app users to select multiple pronouns
2. Bulls-I	Revised	Users specify names for their body parts used throughout the app	Revised so that users can name their own body parts, which were piped into activities throughout the app
3. BottomLine	Original	Users specify their sexual safety practices	Added “almost always” or “rarely” options Added the definitions of condom and dental dam
4. Underwear Personality Quiz	Original	Icebreaker Introduce app user to characters	Added age, hobbies, romantic identity, and sexuality descriptions (eg, Tommy is polyamorous), race or ethnicity descriptions (eg, Nico is biracial), and gender identity for each character (eg, as trans men, transmasculine, and nonbinary) Added sprouting facial hair and acne to characters Added weight to a character to make them plus size (eg, Art is larger)
5. P’s On-Again Off-Again BottomLine	Original	Introduce P, Nico, and P’s new boyfriend Introduce the sexual safety dilemma	Updated informal language and lingo, as old language was viewed as being outdated Added emojis to conversations to reflect common usage
6. Sexy Settings	Revised	Educate about the impact sexual settings may have on sexual safety practices	Allowed users to select multiple answers for each setting Added blurb about statutory consent and public sex laws Emphasized discussion of safety level Added sex work scenarios
7. Going Downhill Fast	Original	Educate about the impact substance use may have on sexual safety practices	Updated language around Viagra and Cialis to be clear the use of it is not studied in transmasculine youth Added sentence about how drugs may be abused to cope with emotional distress Added more current street names for drugs (eg, Rush for poppers)
8. Move Up, Move Back	Original	Educate about disadvantage and privilege in society and how it can impact sexual and safety practices	Removed mother, father, brother, and sister and used more generalized and gender-neutral family terms Added questions about gender and gender expression Added a question about the incarceration of family members Add half step for people who may have an invisible disability Added weight as a marker of status in society
9. HIV True/False	Revised	Educate about HIV and HIV prevention	Shortened text throughout this activity Added information about injectable PrEP^b^ Included information about how to get PrEP for free or at a low cost Included information about the transmasculine need to take PrEP consistently for 21 days for it to reach maximum effectiveness
10. Jeopar-T	Added	Educate and address misinformation and myths about transmasculine health	Adapted for transgender men and transmasculine health Included information about different methods of taking testosterone Included information about birth control options Included information that specified testosterone is not a form of birth control Included language about potential benefits and challenges in early stages of gender-affirming hormone therapy like acne and gender euphoria Added alternatives to binders, such as high-impact sports bras (Tomboy X), that can be worn while doing physical activity or swimming
11. Checking In On Your BottomLine	Original	Check in about BottomLine	No additional changes
12. P Learns About Safer Sex	Original	Educate on healthy ways to cope with difficult emotions	Removed P on the way to the clinic video because it did not resonate with the lived experience of young transgender men and transmasculine YAB^c^ members Repurposed images from the video to describe a more salient scenario: how P feels upset with himself for not using condoms during sex
13. Tackling Testing	Revised	Educate about HIV or STI^d^ testing and PrEP	Split animation into shorter videos, including a short video addressing reproductive health Included potential pregnancy scenario as part of the testing visit Updated provider language to more current HIV testing practices Added captions to videos Added information about what youth can do to talk about safe sex if they do not have a trusted adult to speak with
14. Spread Out	Original	Educate about the chance of HIV exposure based on sexual act	Changed activity name from “Well Hung???” to “Spread Out” Distinguished between front hole sex and anal sex Spelled out acronyms of STIs
15. 12 Steps to Effective Condom Use	Original	Educate about how to properly use an external condom	Added 12 steps to effective internal condom use Updated paragraphs after internal and external condom use lists
16. Safer Injection	Added	Educate about how to safely inject hormones	Adapted to include information about steps for safely injecting testosterone
17. Checking In On Your BottomLine Again	Original	Check in about BottomLine	No additional changes
18. Disclosure and Safety	Added	Provide information on how to safely disclose trans status to sexual and romantic partners	Adapted for salience to transmasculine youth Centered transmasculine youth and young transgender men experience and decentered potential cisgender partner reactions in the activity Emphasized that disclosing is not “a big deal” and can be straightforward Emphasized the need to disclose in a safe place (trusted public settings, around others they trust, or digitally) if app user chooses to disclose Added for app users to be alert and relaxed before disclosure Settled on using “direct, assertive, and honest” language when disclosing Made activity interactive by creating multiple potential response panels from a partner app users work through Included partner disclosure of HIV status and antiviral usage to remain undetectable
19. Red Flag, Green Flag	Added	Educate about safer web-based dating	Added gender to each web-based profile Added a picture to each web-based profile Added information about how location features can be turned off on dating apps Added information about how photos can be screenshots on dating apps despite app screenshot blocking Added information about avoiding partners who fetishize trans people Added information about encountering fake dating profiles
20. Peep in Love	Original	Educate about how intense feelings can impact sexual behavior Educate about practicing direct communication	Added minor changes to boyfriend character to gear toward young adults, that is, boyfriend (Rodney) is 17 years and works at a movie theater
21. Healthy Relationships	Added	Educate about healthy and unhealthy relationship dynamics	Described healthy and unhealthy relationship characteristics Added relationship scenario youth could apply characteristics to
22. 4 Ways to Manage Stigma	Original	Educate about different types of coping approaches in stigmatizing scenarios	Adapted to include references to trans men and transmasculine identity as a stigmatized status with related examples
23. Get a Clue!	Original	Educate about responses in unique sexual settings and circumstances	Removed sexual innuendos youth found inappropriate. For example, “COCKtails” changed to “cocktails”
24. Last Time Checking In On Your BottomLine	Original	Check in about BottomLine	No additional changes
25. BottomLine Overview	Original	Review BottomLine	No additional changes

^a^MyPEEPS: Male Youth Pursuing Empowerment, Education, and Prevention around Sexuality.

^b^PrEP: pre-exposure prophylaxis.

^c^YAB: youth advisory board.

^d^STI: sexually transmitted infection.

### Usability Testing and Heuristic Evaluations

A total of 20 young transgender men participated in usability testing and self-identified as Asian (n=1, 5%), Black (n=2, 10%), White (n=11, 55%), and multiracial (n=3, 15%). Usability testing participants who identified as Hispanic or Latino or Latinx (n=5, 25%) were Puerto Rican (n=3, 15%) and Mexican (n=2, 10%). The mean age of usability testing participants was 20.9 (SD 1.94; range 18-25) years and they were largely from the Northeast (n=10, 50%), with the balance of the group from the West (n=5, 25%), South (n=3, 15%), and Midwest (n=2, 10%). Participants rated their perceived usability of the app through the PSSUQ [15] and Health-ITUES [16]. Mean (SD) scores are reported in Table 2. The mean score of the overall PSSUQ was 1.63 (SD 0.65) and the mean score of the Health-ITUES was 4.50 (SD 0.24), reflecting strong user acceptance of the MyPEEPS young transgender men app. The mean usability score in this study was above the cutoff point of 4.32 on the Health-ITUES used to define the usability of health IT tools [17].

A total of 5 usability experts participated in heuristic evaluations. Responses to the 10 heuristics in each round ranged from no usability problem to minor usability problems, with only 1 participant identifying a major usability problem for user control and freedom, which are listed in Table 3. The expert specifically noted that there was no quick way to exit a module or the app in its entirety.

**Table 2:** PSSUQ^a^ and Health-ITUES^b^ scores across end users (n=20).

Measure		Score, mean (SD)
**PSSUQ overall^c^**		1.72 (0.63)
	System usefulness	1.22 (0.57)
	Information quality	2.11 (0.57)
	Interface quality	1.71 (0.55)
**Health-ITUES overall^d^**		4.50 (0.24)
	System impact on daily life	4.54 (0.25)
	Perceived usefulness	4.49 (0.32)
	Perceived ease of use	4.81 (2.16)
	User control	3.83 (0.30)

^a^PSSUQ: Post-Study System Usability Questionnaire.

^b^Health-ITUES: Health Information Technology Usability Evaluation Scale.

^c^Rating score from 1=best to 7=worst (16 items).

^d^Rating score from 1=worst to 5=best (20 items).

**Table 3:** Expert rating of usability of MyPEEPS^a^ young transgender men app guided by Nielsen heuristics.

Nielsen heuristics^b^	Round 1	Round 2	Round 3	Round 4	Round 5	Score, mean (SD)
Visibility of system status	0	2	2	2	2	1.6 (0.89)
Match between system and real world	0	1	0	2	2	1 (1.00)
User control and freedom	2	3	2	2	0	1.8 (1.09)
Consistency and standards	1	2	0	1	0	0.8 (0.84)
Help users recognize, diagnose, and recover from errors	0	0	0	0	0	0 (0)
Error prevention	0	2	2	2	2	1.6 (0.89)
Recognition rather than recall	0	2	0	0	2	0.8 (1.09)
Flexibility and efficiency of use	0	2	0	0	0	0.4 (0.89)
Aesthetic and minimalist designs	0	1	2	0	0	0.6 (0.89)
Help and documentation	0	2	2	2	0	1.2 (1.09)

^a^MyPEEPS: Male Youth Pursuing Empowerment, Education, and Prevention around Sexuality.

^b^Ranging from 0=not a usability problem to 4=usability catastrophe.

### Open-Ended Feedback

#### Overview

Open-ended feedback was organized by the constructs of the PSSUQ: (1) system usefulness, (2) information quality, and (3) interface quality.

#### System Usefulness

Some participants shared that they believe the app could be useful to younger transgender men and transmasculine people. A 19-year-old White nonbinary transgender man (U2) found the information in the app “accurate and very detailed.” He stated that young people would be able to “comprehend it well.” When discussing the Red Flag, Green Flag activity about web-based dating safety, a Black 23-year-old genderqueer transgender man (U19) said: “I like how it describes for people what is safe and what isn’t when it comes to looking at profiles because when I was young I didn’t know.” He went on to say that the activity was “Pretty accurate to what can happen in real life. Simple and wholesome while showing the reality of things.” Several participants conveyed appreciation for the content warnings throughout the app before sensitive material. They also expressed excitement about the trophies that appeared after they completed each activity. A 21-year-old biracial transgender man (U13) indicated that the trophies made him feel “accomplished.” Another participant (U20) described the trophies as “fun.”

#### Information Quality

Several end users expressed that the dialogue between app characters did not align with how young people speak with one another. On the other hand, a 19-year-old White boyflux (someone who feels fluctuating masculinity [18]) transgender man (U10) shared that he felt the information in the app was presented “in a clear way.”

Some participants expressed that they learned new information by viewing the content. Participants U4 and U19 shared: “I didn’t know trans men could take PrEP.”

#### Interface Quality

A 23-year-old Black and Puerto Rican nonbinary man (U17) described the characters as “cute.” On the other hand, there were concerns with interface quality with multiple usability testers criticizing the presence of long blocks of text in the app. A 22-year-old White transgender man (U4) said: “When I see big chunks of text my brain just doesn’t want to read it.”

User navigation difficulties were identified. A 19-year-old Asian genderqueer participant (U1) described the font as small and difficult to read. When discussing the Red Flag, Green Flag activity, a 19-year-old White nonbinary trans man (U2) shared: “I had to figure out where to swipe or how to swipe.” In response, the app text was condensed to eliminate large text blocks and the remaining text was enlarged throughout the app. Red Flag, Green Flag activity instructions were also rewritten for clarity in response to this feedback.

## Discussion

### Principal Findings

MyPEEPS Mobile is among the first mHealth interventions developed to meet the specific needs of young transgender men to reduce HIV risk behaviors. While many of the activities in the original MyPEEPS app were rigorously developed and tested, there was a need to adapt our intervention to meet the specific needs and risk factors among young transgender men and transmasculine youth. The findings from this study describe the adaptation of these activities through feedback from a YAB and expert advisors. Following adaptation of the content, the app underwent a rigorous usability assessment through an evaluation with experts in human-computer interaction and targeted end users.

The primary adaptations of the app were focused on increasing MyPEEPS character relevancy to young transgender men and transmasculine experiences in addition to removing, expanding, and adding activities for the same purpose. These changes are consistent with our prior formative research suggesting that the MyPEEPS Mobile app while resonating with young transgender men with regard to the basic HIV educational information and sexual scenarios, lacked transmasculine specificity in both its narrative content and its graphics [9]. In this adaptation, the character graphics and backgrounds were revised to reflect the realities of young transgender men. These adaptations are essential to ensure that interventions have saliency for the target population [19].

In addition to these adaptations, we made other more substantial ones. This included removing and adding activities; maintaining the underlying curricular components, namely, the underlying social-cognitive theory that framed the intervention; and content on emotion regulation, HIV knowledge, self-efficacy, and behavioral skills within young transgender men–specific social contexts [4]. These include myths specific to young transgender men and transmasculine HIV and pregnancy risk (“Jeopar-T,” activity 10), hormonal needle injection (“Safer Injection,” activity 16), disclosure of trans status to sexual partners (“Disclosure and Safety,” activity 18), choosing potential partners (“Red Flag, Green Flag,” activity 19), and relationship dynamics (“Healthy Relationships,” activity 21), all of which have been emphasized as important to sexual safety among transgender men and transmasculine youth. This content was aligned with a previous HIV prevention intervention for young transgender men [20].

The findings from the usability evaluation suggested that this app is highly usable and perceived as useful to target end users. These are critical factors in ensuring uptake of the app and have been demonstrated as harbingers to behavior change in our previous studies. Furthermore, there was a usability factor that was found to be a critical error. This was addressed prior to the launch of the app in our feasibility trial.

While the inclusion of a diverse group of young transgender men through both a YAB and usability testing was a clear strength, a notable weakness is that no youth younger than 18 years were included in this formative work, which is a limitation given the app’s intended use is for young transgender men aged 15-25 years. Therefore, conclusions suggesting that MyPEEPS will have high usability are limited for those 18 years of age and older. This is also notable since youth younger than 18 years often have added considerations toward privacy and security. For example, parents may see what they are entering into an app in ways that inadvertently out the youth to their parents.

### Limitations

The MyPEEPS Mobile app adapted for young transgender men by a YAB comprised primarily racial and ethnic minority young transgender men from the Northeastern United States and so may not be generalizable across all racial and ethnic groups and areas of the United States. YAB meeting attendance was poor due to an average of 4 out of 11 youth board members attending each session. MyPEEPS Mobile content largely focused on the relationships between young transgender men and cisgender men, which are not fully representative of the broader transmasculine community nor young transgender men sexual networks that could lead to HIV exposure.

### Conclusions

Usability and adaptation findings demonstrate that the MyPEEPS Mobile is highly usable and perceived as potentially useful for targeting HIV risk behaviors in young transgender men and transmasculine youth. This study focused on the adaptation of an existing EBI. A larger randomized controlled pilot study (ClinicalTrials.gov NCT 05424718) is planned, which will assess the feasibility of the app as an HIV prevention intervention for young transgender men and transmasculine youth.

## Abbreviations

EBI: evidence-based intervention
HIPAA: Health Insurance Portability and Accountability Act
Health-ITUES: Health Information Technology Usability Evaluation Scale
mHealth: mobile health
MyPEEPS: Male Youth Pursuing Empowerment, Education, and Prevention around Sexuality
PrEP: pre-exposure prophylaxis
PSSUQ: Post-Study System Usability Questionnaire
REDCap: Research Electronic Data Capture
YAB: youth advisory board

## Data Availability

The data generated and analyzed during this study are available from the corresponding author on reasonable request.
